# Long term Follow up Study of Phone Contacts in Catalonia

**DOI:** 10.1192/j.eurpsy.2022.128

**Published:** 2022-09-01

**Authors:** D. Palao Vidal, I. Parra, A. Cebrià Meca, M. Guinovart, R. Gracia

**Affiliations:** 1 Parc Tauli-University Hospital, Mental Health, Sabadell (Barcelona), Spain; 2 School of Medicine, Universitat Autònoma de Barcelona, Medicine, Cerdanyola del Vallés, Spain; 3 Centro de Investigación Biomédica en Red de Salud Mental (CIBERSAM), Salud Mental, Madrid, Spain; 4 Autonomous University of Barcelona (UAB), Department Of Clinical And Health Psychology, Cerdanyola del Vallès (Barcelona), Spain

**Keywords:** telephone, attempts, Suicide, prevention

## Abstract

We’ll present the Catalan experience of follow up by phone contats of suicide attempters, and the results up to 10 years. We develop a Suicide Prevention Program that started with the EAAD project in 2008 in a 0,5 M people catchment area, later generalized to 7,5 M people through the Catalonia Suicide Risk Code (SRC-Cat). The SRC-cat is a real-time registry of suicide attempts (65% women) that allows immediate attention and telephone follow-up and ensures continuity of care for 12 months. To evaluate the effectiveness of our telephone management plan, we conducted two types of analysis; a) 12-month short-term analysis: non-randomised controlled analysis of suicide reattempts comparing two cities (2007-2008); b) 8-year long-term analysis with the evolution of suicide rates (men and women) between our area, and two other cities (territorial differences and over time from 2010 to 2017). Results: a) the SRC-Cat in our catchment area reduced significantly the proportion of people who re-attempt suicide by 57% over 12 months (from 14% to 6 %); b) we found lower standardized suicide death rate among women in our catchment area (both territorially and over time). Conclusions: a) Short-term telephone management (12-month), ensuring chain of care after hospital discharge, reduces more than 50% the proportion of patients who re-attempt; b) Long-term telephone management (8-year) of suicide attempt survivors over 12 months, significantly reduces suicide deaths in women only (64% of patients in telephone follow-up are women).

**Disclosure:**

No significant relationships.

